# HemoTypeSC, a low-cost point-of-care testing device for sickle cell disease: Promises and challenges

**DOI:** 10.1016/j.bcmd.2019.01.007

**Published:** 2019-09

**Authors:** Obiageli Nnodu, Hezekiah Isa, Maxwell Nwegbu, Chinatu Ohiaeri, Samuel Adegoke, Reuben Chianumba, Ngozi Ugwu, Biobele Brown, John Olaniyi, Emmanuel Okocha, Juliet Lawson, Abdul-Aziz Hassan, Ijeoma Diaku-Akinwumi, Anazoeze Madu, Osita Ezenwosu, Yohanna Tanko, Umar Kangiwa, Ahmed Girei, Yetunde Israel-Aina, Adama Ladu, Perpetua Egbuzu, Usman Abjah, Angela Okolo, Nagihan Akbulut-Jeradi, Maria Fernandez, Frédéric B. Piel, Adekunle Adekile

**Affiliations:** a Centre of Excellence for Sickle Cell Disease Research & Training, University of Abuja, Abuja, Nigeria; b Department of Paediatrics, Federal Medical Centre Keffi, Nasarawa State, Nigeria; c Department of Paediatrics, Obafemi Awolowo University Teaching Hospital, Ile Ife Osun State, Nigeria; d Department of Haematology, Federal Teaching Hospital, Abakaliki, Ebonyi State, Nigeria; e University College Hospital, Ibadan, Oyo State, Nigeria; f Nnamdi Azikiwe University Teaching Hospital, Nnewi, Anambra State, Nigeria; g Zankli Medical Centre, Abuja, Nigeria; h Department of Haematology, Ahmadu Bello University, Zaria, Kaduna State, Nigeria; i Department of Paediatrics, Lagos State University Teaching Hospital, Lagos, Nigeria; j Department of Haematology, University of Nigeria Teaching Hospital, Enugu, Nigeria; k Department of Haematology, Federal Medical Centre Birnin-Kebbi, Kebbi State, Nigeria; l Department of Haematology, Federal Medical Centre Gombe, Gombe State, Nigeria; m Department of Paediatrics, University of Benin Teaching Hospital, Benin Edo State, Nigeria; n University of Maiduguri Teaching Hospital, Maiduguri, Borno State, Nigeria; o Federal Medical Centre, Asaba, Delta State, Nigeria; p Advanced Technology Company, Kuwait; q Department of Epidemiology & Biostatistics, School of Public Health, Imperial College, London, UK; r Department of Paediatrics, Kuwait University, Kuwait

**Keywords:** Low-cost, Point-of -care -test devise, Sickle cell disease, Newborn screening, HemoTypeSC

## Abstract

**Background:**

Sickle cell disease (SCD) is a neglected burden of growing importance. >312,000 births are affected annually by sickle cell anaemia (SCA). Early interventions such as newborn screening, penicillin prophylaxis and hydroxyurea can substantially reduce the mortality and morbidity associated with SCD. Nevertheless, their implementation in African countries has been mostly limited to pilot projects. Recent development of low-cost point-of-care testing (POCT) devices for sickle haemoglobin (HbS) could greatly facilitate the diagnosis of those affected.

**Methods:**

We conducted the first multi-centre, real-world assessment of a low-cost POCT device, HemoTypeSC, in a low-income country. Between September and November 2017, we screened 1121 babies using both HemoTypeSC and HPLC and confirmed discordant samples by molecular diagnosis.

**Findings:**

We found that, in optimal field conditions, the sensitivity and specificity of the test for SCA were 93.4% and 99.9%, respectively. All 14 carriers of haemoglobin C were successfully identified. Our study reveals an overall accuracy of 99.1%, but also highlights the importance of rigorous data collection, staff training and accurate confirmatory testing. It suggests that HPLC results might not be as reliable in a resource-poor setting as usually considered.

**Interpretation:**

The use of such a POCT device can be scaled up and routinely used across multiple healthcare centres in sub-Saharan Africa, which would offer great potential for the identification and management of vast numbers of individuals affected by SCD who are currently undiagnosed.

**Funding US:**

Imperial College London's Wellcome Trust Centre for Global Health Research (grant #WMNP P43370).

**Research in context**

**Evidence before this study**: Several POCT devices for sickle cell disease have already shown promising results in laboratory settings and in a few small pilot studies.

**Added value of this study**: Our study demonstrates both the feasibility of using a low-cost POCT across multiple health centres in sub-Saharan Africa, and the high sensitivity and specificity of the device in low- and middle-income settings.

**Implications of all the available evidence**: Such devices could considerably facilitate testing for sickle cell disease in resource poor settings. This could have a considerable impact on high-prevalence countries if the results can be accurately recorded and patients can be followed up to prevent severe long-term chronic complications.

Alt-text: Unlabelled Box

## 1 Introduction

Sickle cell disease (SCD) is an autosomal recessive inherited disorder affecting the structure of normal haemoglobin (HbA). Homozygotes (HbSS genotype) have the most common form of the disease, also referred to as sickle cell anemia (SCA) [1]. The abnormal HBB S gene can also be co-inherited with another abnormal beta globin gene, resulting in other genotypes of the disease like HbSC, HbSE and HbSO_Arab_ and with a β-thalassemia allele, resulting in HbS-ß^0^-thalassemia or HbS-ß^+^-thalassemia depending on how much of the ß chain is synthesized. Heterozygotes or carriers (HbAS) have sickle cell trait. They are usually clinically normal and benefit from a high level of protection against severe malaria, hence the highest frequencies of the gene are observed in malaria-endemic regions. >300,000 infants are born annually worldwide with SCD, the vast majority (~75%) of which occurs in sub-Saharan Africa (SSA) [2]. At a national level, the burden of SCD is highest in Nigeria due to its large population (196 million in 2018), a high birth rate (36.9 births/1000 population) and a conservative SCA birth prevalence of 2% [[3], [4], [5]].

SCD is associated with a high under-five mortality (50–90%) [6] and morbidity characterized by chronic haemolysis and recurrent painful vaso-occlusion [7]. Osteonecrosis, acute chest syndrome and stroke are also common [7]. In addition, adult patients are prone to chronic organ failure [8]. Although SCD also remains a substantial cause of morbidity from acute complications and chronic organ damage in high-income countries, significant improvements have been reached in relation to survival with >90% of patients now expected to live to adulthood [9,10]. A number of medical advances are responsible for this, including early diagnosis, comprehensive care and penicillin prophylaxis. In the US and the UK, universal newborn screening has long been implemented [11]. However, there is currently no country in SSA where universal newborn screening is practiced, although several pilot programs have shown varying degrees of success [12].

One of the main challenges in SSA is that the majority of the population lives in rural areas and does not have access to healthcare [3]. The current laboratory diagnosis of SCD is based on Hb electrophoresis, iso-electric focusing, high performance liquid chromatography (HPLC), mass spectrometry and molecular techniques. All these are capital intensive and require highly-trained technical personnel and a stable power source, which are not readily available in most of the resource-poor SSA countries. There is therefore a need for inexpensive, reliable, easy to use, point-of-care testing (POCT) devices with high specificity and sensitivity in the discrimination of the different Hb phenotypes. Such a device would have many advantages including I) ease of use by local staff; ii) rapid results delivery, enabling prompt notification of the patients and counselling of their relatives when necessary; iii) the possibility to use the test in remote sites leading to early diagnosis and to reductions in mortality and morbidity through the use of therapeutic interventions, especially penicillin prophylaxis and anti-malarials.

Several POCT devices for SCD have recently been developed based on different diagnostic principles including differential erythrocyte density [13], differential mobility of Hb S and Hb A through filter paper [14] and a polyclonal antibody-based capture immunoassay [15]. All of these have their limitations either because they require instrumentation as an integral part of the procedure to achieve maximum specificity and sensitivity or because of limited accuracy [16]. More recently, a new POCT (HemoTypeSC) was developed, based on monoclonal antibodies (MAb) that differentiate normal adult haemoglobin (HbA), sickle haemoglobin (HbS) and haemoglobin C (HbC) [17]. In a competitive enzyme-linked immunosorbent assay, each MAb bound only its target with <1.0% cross-reactivity. In a laboratory setting, HemoTypeSC was shown to be 100% accurate in identifying the correct Hb phenotype in a group of 100 whole blood samples from individuals with common relevant Hb phenotypes [17]. These antibodies are blind to haemoglobin F (HbF) so even newborns with elevated HbF and low levels of HbA or HbS can be accurately diagnosed.

Here, we have evaluated the overall accuracy, specificity and sensitivity of HemoTypeSC in identifying Hb phenotypes (AA, AS, AC, SS, SC, and CC) across multiple Nigerian primary healthcare centres in a real-life, field setting, in order to scope its potential for large-scale use throughout Nigeria and other SSA countries.

## 2 Methods

### 2.1 Study design

**Fig. 1:**
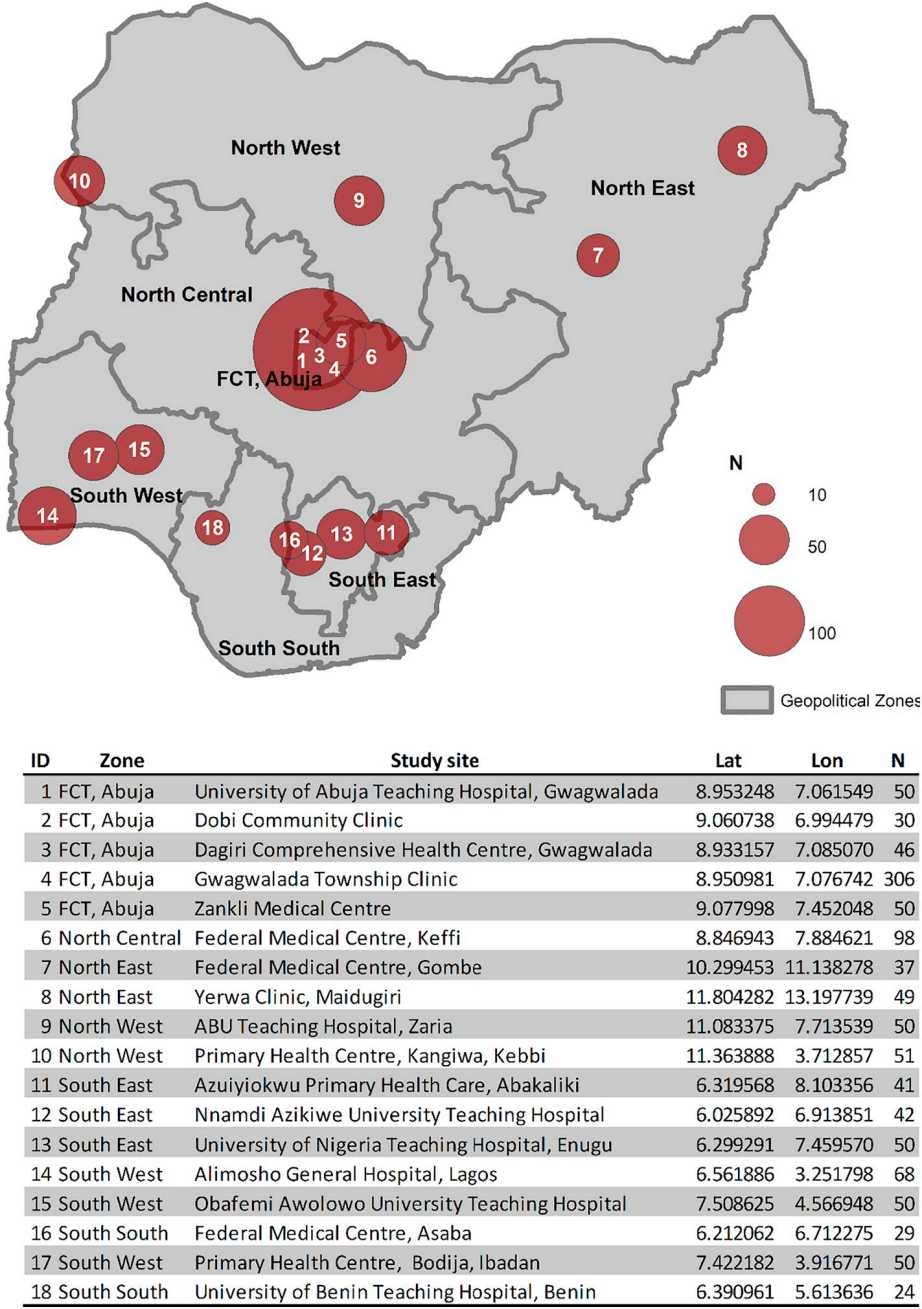
Map and summary table of the 18 participating centres and numbers of patients screened. Lat: latitude; Lon: longitude; N: number of individuals tested.

The primary aim of the study was to evaluate the clinical accuracy of HemoTypeSC for testing newborns and infants less than one year old across a range of urban, semi-urban and rural health centres in Nigeria. Power calculations based on a 95% clinical sensitivity, 0.05 statistical precision and birth prevalence of HbS of 10% suggested a minimum overall sample size of 700 individuals or 100 individuals per geopolitical zone. Ethical clearance was obtained from the University of Abuja Teaching Hospital Ethics Committee, prior to commencement of the study. >1100 were ultimately tested at 18 primary care centres affiliated to collaborating centres of the Sickle Cell Support Society of Nigeria (SCSSN, http://scsn.com.ng/) across the six geopolitical zones of the country (Fig. 1). The sampling was carried out between September and December 2017. All babies with a history of blood transfusion were excluded. The parents of all eligible babies presenting in the participating centres were approached for testing. Informed signed consent was obtained. The date of birth, age and sex of each individual screened were recorded. The “Standard Precautions” protocol developed by the US Centers for Disease Control and Prevention was followed throughout the sample collection and testing to prevent infection when working with human blood samples [17].

### 2.2 Staff training

At least one haematologist, one nurse, one biochemist and one community health extension worker were trained by the manufacturer of the device, Silver Lake Research Corporation, to use the HemoTypeSC for SCD testing through lectures, video presentations and hands-on training. The training included properly performing the test and correctly interpreting the results of the test. Each health worker was then evaluated. Additional training was provided for staff at each centre, until 100% of the training samples were correctly identified.

### 2.3 Storage

The HemoTypeSC test kits containing the lateral flow assay (LFA) test strip, a transfer pipette, a sample cup, and a volumetric inoculation loop were stored at room temperature. Typical temperature and humidity ranges during the months of the study are 19.3–35.50 °C and 38–81%, respectively in Abuja (20.1–38 °C and 24–64% in Maiduguri; 22.3–33.0 °C and 62–86% in Enugu; 22.1–34.8 °C and 61–85% in Ibadan) (*personal communication* from the Nigerian Meteorological Agency). HemoTypeSC is considered to be stable in high heat and does not require refrigeration.

### 2.4 Sampling and testing

**Fig. 2:**
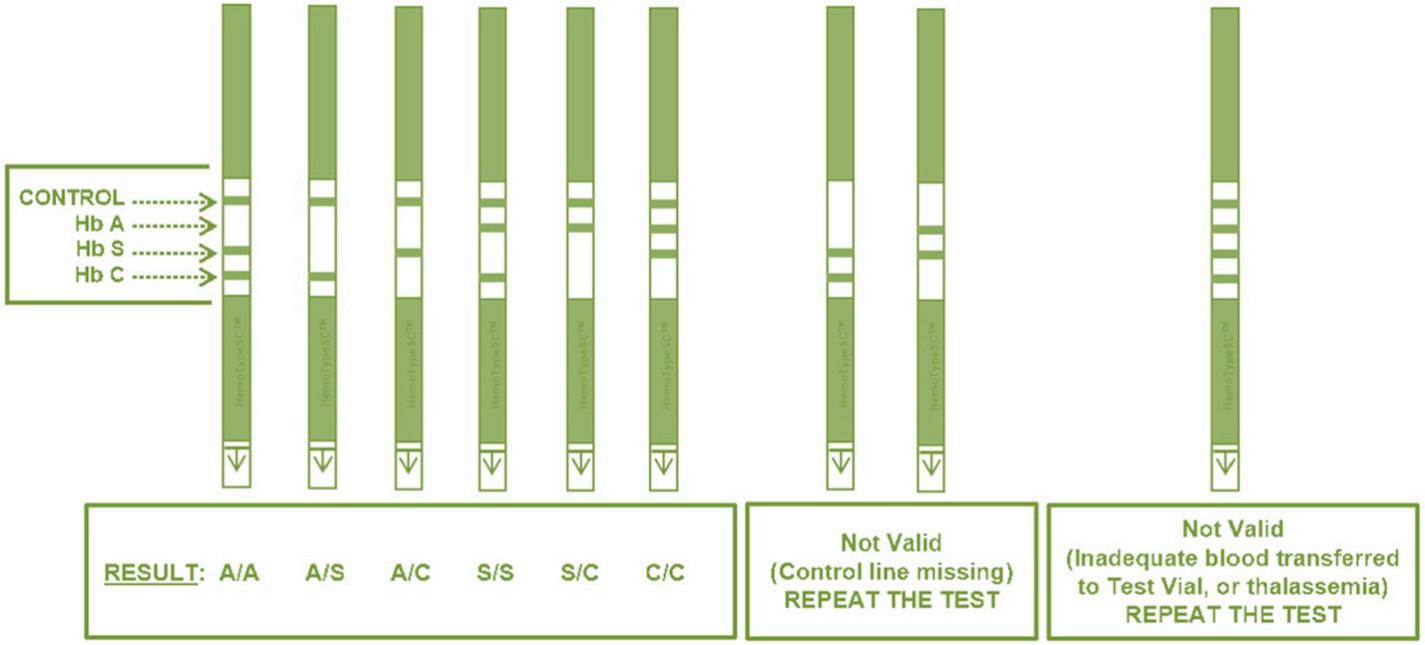
Manufacturer's chart to assist with the interpretation of the HemoTypeSC tests for sickle cell disease. Lines appear for the control and the haemoglobin variants not identified in the sample tested.

Blood samples from babies six weeks and below were drawn by heel-prick, while those from older infants were collected by finger-prick. Approximately 1 μl of blood was absorbed into the HemoTypeSC blood sampling device absorbent pad for testing, and additional blood from the same blood draw was sampled by capillary into labelled filter paper cards supplied by the Association of Public Health Laboratories (https://www.aphl.org/). Samples were tested with HemoTypeSC on the day of collection in each of the local participating centre. The tests were performed strictly according to the manufacturer's instructions and interpreted based on a reference chart provided by the manufacturer (see Fig. 2). Anonymized results were shared in real time through a secure social media platform. Blood spots were air dried and shipped within a week of sampling to the national newborn screening reference laboratory in Keffi, Nassarawa State, for HPLC testing. Samples were blindly tested according to standard methods using the Biorad nbs variant machine. Clinical control samples of previously-diagnosed AA, AS, SS and SC subjects/patients were included with each batch of HemoTypeSC and HPLC tests to assess the performance of these techniques. Results from HemoTypeSC and HPLC were then returned to the University of Abuja Centre of Excellence in Sickle Cell Disease Research and Training (CESRTA, https://cesrta.uniabuja.edu.ng/) for analysis. Samples which produced discordant results were further tested by DNA Sanger sequencing, provided that sufficient blood was still available. DNA was extracted at the Institute of Virology, Abuja using the protocol of QIAamp DNA blood mini kit, before being shipped to Kuwait for Sanger sequencing at the Advanced Technology Company (http://www.atc.com.kw/). The evaluation was conducted in accordance with the guidelines for evaluation of qualitative tests as set forth by the Clinical and Laboratory Standards Institute (CLSI, https://clsi.org/).

### 2.5 Assessment

The sensitivity, specificity, positive and negative predictive values, and overall accuracy of HemoTypeSC compared to “gold standard” HPLC and DNA sequencing were independently calculated by the CESTRA team (ON) and an independent expert (FBP). Sensitivity was defined as 100% × TP/(FN + TP) specificity as 100% × TN / (FP + TN), positive predictive value as 100% × TP / (TP + FP), negative predictive value as 100% × TN / (TN + FN) and overall accuracy as (prevalence × sensitivity) / (1 − prevalence)(specificity), where TP = number of true positive events, FP = number of false positive events, and TN = number of true negative events [18]. The allele frequency of HbS was also calculated for each of the participating centres.

## 3 Results

HemoTypeSC was evaluated in a total of 1121 newborns and infants from 18 participating centres across Nigeria. Of these, 552 (49.2%) and 569 (50.8%) were females and males, respectively. The age distribution of the individuals screened is summarized in Table 1.

**Table 1:** Age distribution of the newborns and infants tested for sickle cell disease across the 18 Nigerian participating centres.

Age	N	%
0–1 day	37	3.3%
2 days–<1 month	194	17.3%
1–<3 months	308	27.5%
3–<6 months	244	21.8%
6–≤9 months	223	19.9%
>9 months–1 year	57	5.1%
Unavailable	58	5.1%
	1121	100%

Overall, the HemoTypeSC tests identified 834 HbAA (74.3%), 257 HbAS (22.9%), 16 HbSS (1.4%), and 14 HbAC (1.2%). The test cannot differentiate between HbSS and sickle-β^0^-thalassemia. No HbCC or HbSC were identified. Details per centre and allele frequencies are presented in Table 2. HPLC results, which are considered as the “gold standard”, showed a total of HbAA as 835 (74.4%), HbAS 253 (22.5%), HbSS 13 (1.1%), HbAC, 14 (1.2%), HbSC, 1 (0.08%) and others, 5 (0.4%). Discordant results were found for a total of 30 samples (Table 3), which were sent for DNA analysis. For three samples from Keffi, no HPLC results could be obtained and the DNA analysis confirmed the output of HemoTypeSC. Seven of the discordant samples appeared to reflect simple data entry errors (e.g. “A3” instead of “AS”). One of these samples was identified as HbSFE disease by HPLC, matching the diagnosis of SCD made by HemoTypeSC and DNA analysis. Of the 20 remaining discordant samples, two samples returned different results for the three testing methods (1 HbAA, 1 HbAS and 1 HbSS). DNA sequencing validated HPLC results for ten discordant samples (including seven from Gombe center). In the eight remaining discordant samples, we found that the DNA analyses confirmed the diagnosis from HemoTypeSC over the results of the HPLC, which suggest that four carriers and three individuals with SCD would have been missed if HPLC was the only testing method; the last discordant sample being identified as HbSC by HPLC and HbSS by HemoTypeSC and DNA testing.

**Table 2:** Genotypes and allele frequencies identified by HemoTypeSC in the 18 Nigerian participating centres, and genotypes expected according to Hardy-Weinberg Equilibrium (HWE).

ID	Name	N	Genotype				Allele frequency (%)		Expected (HWE)		
			AA	AS	SS	AC	S	C	AA	AS	SS
1	Abuja	50	41	9	0	0	9.00	/	41	9	0
2	Dobi	30	22	6	1	1	13.33	/	23	8	1
3	Dagiri	46	35	10	0	1	10.87	/	37	10	1
4	Gwagwalada	306	224	78	4	0	14.05	0.58	226	86	6
5	Zankli	50	34	15	1	0	17.00	/	34	17	1
6	Keffi	98	55	18	1	1	10.20	/	79	20	1
7	Gombe	37	29	8	0	0	10.81	/	29	8	0
8	Maidugiri	49	39	10	0	0	10.20	/	40	10	1
9	Zaria	50	33	13	2	2	17.00	/	34	17	1
10	Kebbi	51	36	14	0	0	13.73	/	38	14	1
11	Abakaliki	41	32	9	0	0	10.98	/	32	9	0
12	Nnewi	42	34	8	0	0	9.52	/	34	8	0
13	Enugu	50	45	5	0	0	5.00	/	45	5	0
14	Lagos	68	52	9	3	4	11.03	/	54	15	1
15	Ile-Ife	50	33	14	2	1	18.00	/	34	18	2
16	Asaba	29	21	8	0	0	13.79	/	22	8	1
17	Ibadan	50	37	11	0	2	11.00	/	40	11	1
18	Benin	24	15	7	1	1	18.75	/	16	9	1
	Total	1121	817	252	15	13	12.58	0.58	857	282	18

**Table 3:** Results from HemoTypeSC, HPLC and DNA testing for the 30 discordant samples identified amongst the 1121 samples tested. Discordant results are shown in bold. The last two samples shown were discordant between the three testing methods and could not be further tested.

N	Centre	HemoTypeSC	HPLC*	DNA
1	Keffi	AS	***	AS
2	Keffi	SS	***	SS
3	Keffi	AS	***	AS
4	Abakaliki	AS	A3 [AS]	AS
5	Abuja, FCT	SS	F3EA [FSEA]	SS
6	Asaba	AS	A3 [AS]	AS
7	Lagos	AS	AE3 [AES]	AS
8	Nnewi	AS	A3 [AS]	AS
9	Nnewi	AS	A3 [AS]	AS
10	Zaria	SS	3FE [SFE]	SS
11	Abuja, FCT	**AS**	AA	AA
12	Asaba	**AS**	AA	AA
13	Benin	**AS**	AA	AA
14	Gombe	**AA**	AS	AS
15	Gombe	**AA**	AS	AS
16	Gombe	**AA**	AS	AS
17	Gombe	**AA**	AS	AS
18	Gombe	**AS**	SS	SS
19	Gombe	**AA**	AS	AS
20	Gombe	**AA**	AS	AS
21	Abuja, FCT	SS	**AA**	SS
22	Abuja, FCT	AS	**AD**	AS
23	Abuja, FCT	AS	**AD**	AS
24	Asaba	AS	**AA**	AS
25	Ife-Ife	SS	**SC**	SS
26	Ife-Ife	SS	**AC**	SS
27	Lagos	AS	**AA**	AS
28	Lagos	SS	**DF**	SS
29	Abuja, FCT	**SS**	**AS**	**AA**
30	Maiduguri	**AA**	**AS**	**SS**

*** Symbols for noise when the HPLC machine was not able to identify the Hb variant in the sample even when repeated.

Due to the particularly high rate (19%) of discordant results observed in one of the participating centres (Gombe), sensitivity, specificity, positive predictive value (PPV), negative predictive value (NPV) and overall accuracy of HemoTypeSC compared to HPLC were calculated for both the total 1121 samples and a subset excluding the 37 samples from Gombe (n = 1084) (Table 4A & B). HemoTypeSC successfully identified all the HbAC cases. While all the indicators, apart from the PPV for HbSS were high when all the samples were considered, the removal of potentially mis-read samples in one site and the additional validation provided by DNA analyses resulted in optimal accuracy for HbSS.

**Table 4:** Genotypes identified by HemoTypeSC compared to those obtained by HPLC (“gold standard”) for the overall 1121 samples (A) and for a subset excluding the 37 samples from one site in which the discordant rate was particularly high (B), and compared to those obtained by HPLC and DNA analysis with the exclusion of 2 completely discordant samples for all study sites (C) and all sites excluding Gombe (D). Sensitivity, specificity, positive predictive value (PPV), negative predictive value (NPV) and overall accuracy are also presented. Others include samples with HbD (1 HbAD, 1 HbADF and 1HbADFS) and HbE (1 HbSE).

A		HemoTypeSC				Total	B		HemoTypeSC				Total
		AA	AS	SS	AC				AA	AS	SS	AC	
HPLC	AA	826	8	1	0	835	HPLC	AA	803	8	1	0	812
	AS	8	244	2	0	254		AS	2	237	2	0	241
	SS	0	1	9	0	10		SS	0	0	9	0	9
	AC	0	0	1	14	15		AC	0	0	1	14	15
	SC	0	0	1	0	1		SC	0	0	1	0	1
	Other	0	4	2	0	6		Other	0	4	2	0	6
	Total	834	257	16	14	1121		Total	805	249	16	14	1084

	Sensitivity	0.989	0.961	0.900	0.933			Sensitivity	0.989	0.983	1.000	0.933	
	Specificity	0.972	0.985	0.994	1.000			Specificity	0.993	0.986	0.993	1.000	
	PPV	0.990	0.949	0.563	1.000			PPV	0.998	0.952	0.563	1.000	
	NPV	0.969	0.988	0.999	0.999			NPV	0.968	0.995	1.000	0.999	
	Accuracy					0.975		Accuracy					0.981


C		HemoTypeSC				Total	D		HemoTypeSC				Total
		AA	AS	SS	AC				AA	AS	SS	AC	
HPLC + DNA	AA	826	3	1	0	830	HPLC + DNA	AA	803	3	1	0	807
	AS	6	249	0	0	255		AS	0	242	0	0	242
	SS	1	1	15	0	17		SS	1	0	15	0	16
	AC	0	0	0	14	14		AC	0	0	0	14	14
	SC	0	0	0	0	0		SC	0	0	0	0	0
	Other	1	4	0	0	5		Other	1	4	0	0	5
	Total	834	257	16	14	1121		Total	805	249	16	14	1084

	Sensitivity	0.995	0.976	0.882	1.000			Sensitivity	0.995	1.000	0.938	1.000	
	Specificity	0.973	0.991	0.999	1.000			Specificity	0.993	0.992	0.999	1.000	
	PPV	0.990	0.969	0.938	1.000			PPV	0.998	0.972	0.938	1.000	
	NPV	0.986	0.993	0.998	1.000			NPV	0.986	1.000	0.999	1.000	
	Accuracy					0.985		Accuracy					0.991

## 4 Discussion

Given the high burden of SCD in SSA and the clear evidence from high-income countries of the substantial benefits of relatively cheap interventions such as early diagnosis, penicillin prophylaxis and hydroxyurea, it is essential to scale up efforts to adopt these strategies in SSA. Unfortunately, such efforts are impeded by the lack of accessibility to healthcare facilities for large portions of the population of these countries. A reliable, easy-to-use, cheap POCT device has the potential to considerably facilitate the identification of individuals with SCD in Nigeria and other countries in which the SCD prevalence is high.

Our study provides the first national study of a POCT device, HemoTypeSC, in an African country aiming to assess its sensitivity, specificity, PPV and NPV in primary care settings. Our results support the laboratory findings previously published, suggesting a sensitivity and specificity of 100% for HbS and HbC in ideal conditions. Nevertheless, it also highlights several important challenges in terms of staff training, particularly considering the counter-intuitive reading of the test, and of good practices when validating the results with a “gold standard” method. The high rate of discordant results identified in one of the study sites suggests human error in reading the results, thus underscoring the ongoing need for adequate training and quality control.

The management of SCD has to devolve around primary care, with emphasis on programs that use simple, affordable technology and reach a large proportion of the community [19]. To this end, the SCSSN has instituted training programs for community health workers in Nigeria for their involvement in SCD management. The curriculum incorporates basic understanding of SCD, genetic counselling, health maintenance, strategies to prevent complications and identification of patients that need referral to secondary or tertiary centres.

HPLC is often considered as one of the “gold standard” methods for SCD screening. Because it relies on expensive equipment, highly trained staff, the availability of reagents and other disposables, and sustained electric power, it is unlikely to be a viable option for wide-scale screening in a resource-poor environment. In addition, our study suggests that this method is also prone to errors, which may be human or due to problems with reagents and other supplies. The fact that discordant results from the three screening methods used in this study – HemoTypeSC, HPLC and DNA testing – were found in 2 samples confirms the importance of both collecting sufficient blood samples for retesting and using the best available confirmation method locally.

So far, low-income, high-burden countries have not been able to go beyond pilot programs due to the high cost of establishing sustained newborn screening programs which involve expensive, elaborate equipment that are not readily affordable or available, particularly outside large urban centres. The present study, which built on an existing public health program of immunization clinics in the Federal Capital and the six geopolitical zones of Nigeria, suggests that HemoTypeSC presents many promising characteristics, including a high sensitivity and specificity combined with a relatively low price, which could considerably improve the diagnosis of SCD across large portions of the population of SSA, and possibly India. Several POCT devices are now available and, while many of them have demonstrated impressive specificity and sensitivity in differentiating the various Hb phenotypes, they all have their strengths and limitations. We had previously tested another POCT, SickleSCAN, which also demonstrated impressive accuracy, specificity and sensitivity in detecting HbSS and HbSC [20]. The choice of device to adopt in any center may eventually depend on ease of use and cost. Indeed, if cost can be brought down sufficiently, POCT device may be preferable to HPLC or IEF as the method of choice for mass screening for SCD in resource-poor populations.
